# Surgical strategy for an adult patient with a catecholamine-producing ganglioneuroblastoma and a cerebral aneurysm: a case report

**DOI:** 10.1186/s40792-018-0529-x

**Published:** 2018-09-17

**Authors:** Hiroyuki Kumata, Ryuichi Nishimura, Chikashi Nakanishi, Chihiro Inoue, Yuta Tezuka, Hidenori Endo, Shigehito Miyagi, Teiji Tominaga, Michiaki Unno, Takashi Kamei

**Affiliations:** 1 Department of Surgery, Graduate School of Medicine, Tohoku University, 1-1 Seiryou-machi, Aobaku, Sendai, 980-8574 Japan; 2 Department of Pathology, Graduate School of Medicine, Tohoku University, 1-1 Seiryou-machi, Aobaku, Sendai, 980-8574 Japan; 3 Division of Nephrology, Endocrinology and Vascular Medicine Tohoku University Graduate School of Medicine, 1-1 Seiryou-machi, Aobaku, Sendai, 980-8574 Japan; 4 Department of Neurosurgery, Tohoku University Graduate School of Medicine, 1-1 Seiryou-machi, Aobaku, Sendai, 980-8574 Japan

**Keywords:** Ganglioneuroblastoma, Cerebral aneurysm, Lumbar catheter

## Abstract

**Background:**

Ganglioneuroblastomas, particularly those that produce catecholamine, are extremely rare in adults. Here, we report an interesting surgical case of an adult patient with a catecholamine-producing ganglioneuroblastomas in her adrenal gland, suspected to be a pheochromocytoma, and with a cerebral aneurysm.

**Case presentation:**

The patient was a 73-year-old woman under treatment for hypertension. During a health check-up, a cystic retroperitoneal tumor was incidentally found in the superior pole of her right kidney. Her blood adrenaline level was slightly elevated, and her urinary adrenaline, noradrenaline, and dopamine levels were above the upper reference limits. In addition, 24-h urinary excretion of metanephrine, normetanephrine, and vanillylmandelic acid were all increased. 123I-Meta-iodobenzylguanidine scintigraphy showed an abnormal accumulation of the marker in the cyst wall. She was, therefore, diagnosed with a pheochromocytoma and scheduled for tumor resection. However, preoperatively, 8-mm-diameter cerebral aneurysm was incidentally found in her basilar artery. This required careful preoperative discussion. The aneurysm was difficult to approach and treat, and based on its position, shape, and size, the risk of rupture was low. Because hypertension is a major risk factor for aneurysmal rupture, we decided to proceed with the tumor resection. A lumbar catheter was placed to monitor the cerebral aneurysm for intraoperative rupture, and her transcranial motor-evoked potential and somatosensory-evoked potentials were monitored to track her intraoperative neurological function. During surgery, we carefully monitored fluctuations in blood pressure and resected the tumor with minimal mobilization. Postoperatively, head computed tomography confirmed that there was no sign of rupture. Histopathologically, the tumor was diagnosed as a catecholamine-producing ganglioneuroblastoma. The postoperative course was good, and the patient’s blood pressure improved.

**Conclusions:**

Careful perioperative management is needed for a patient with both a catecholamine-producing tumor and cerebral aneurysm.

## Background

Ganglioneuroblastomas (GNBs) are derived from neural crest cells and comprised a mixture of neuroblasts and ganglion cells [1]. Some GNBs produce catecholamines [2], making it difficult to distinguish them from paragangliomas by using catecholamine dynamics, such as the 24-h urinary excretion of catecholamines and their metabolites, or by 123I-meta-iodobenzylguanidine (123I-MIBG) scintigraphy. Surgical resection is the primary choice for both types of tumor; however, intraoperative blood pressure fluctuations present a high risk of complications, particularly in patients with abnormal hypertension. If the patient has a cerebral aneurysm, an even more careful strategy is needed. Here, we report an interesting surgical case of an adult patient with a catecholamine-producing GNB in her adrenal gland, leading us to suspect it to be a pheochromocytoma, as well as a cerebral aneurysm.

## Case presentation

**Fig. 1:**
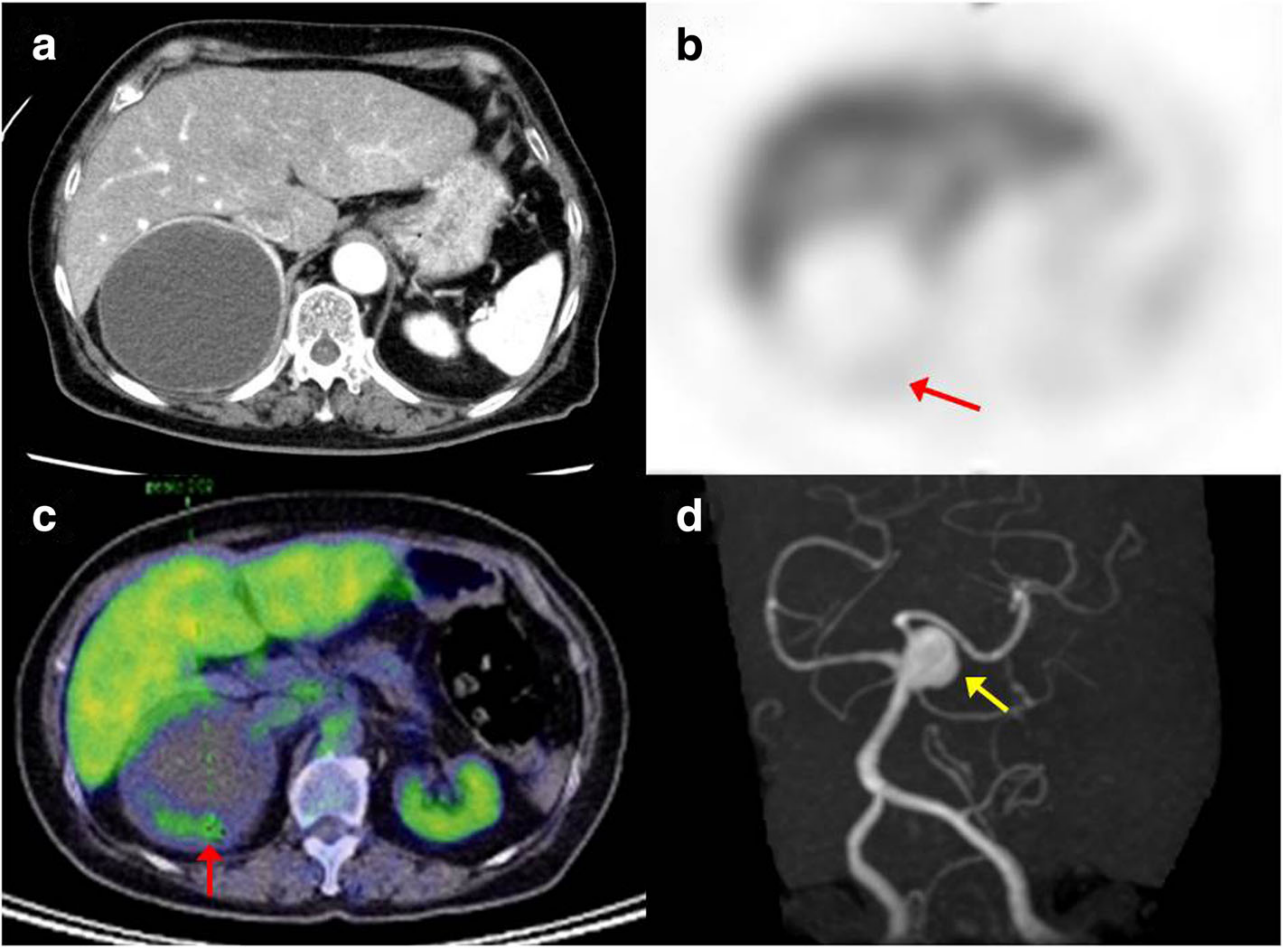
Preoperative findings. **a** Enhanced computed tomography showed a cystic retroperitoneal tumor with a maximum diameter of 88 mm within the right adrenal gland. It had a walled nodule; a contrast effect was observed in the early phase along the cyst wall, including the nodule. **b** ^123^I-meta-iodobenzylguanidine scintigraphy and **c** positron emission tomography with 2-deoxy-2-[fluorine-18] fluoro-D-glucose/computed tomography examination, showing the abnormal accumulation of the markers along the cyst wall (red arrow). **d** Magnetic resonance angiography showing an 8-mm-diameter cerebral aneurysm at the origin of the left anterior cerebral artery (yellow arrow)

During a routine health check-up of a 73-year-old woman, abdominal ultrasonography incidentally revealed a retroperitoneal tumor with a maximum diameter of approximately 80 mm at the upper pole of her right kidney. She was admitted to our institution for examination of the tumor. The patient had been taking medication for hypertension, and her blood pressure had been maintained at 120–130 mmHg with 20-mg/day nifedipine and 2.5-mg/day carvedilol. She had no notable abnormal findings in her general biochemistry, complete blood count, or the coagulation test. Various tumor markers, including carcinoembryonic antigen, carbohydrate antigen 19-9, squamous cell carcinoma-related antigen, and carbohydrate antigen 125, levels were also within normal ranges. Her blood noradrenaline and dopamine levels were within normal ranges, but her adrenaline level was elevated at 0.12 ng/ml and the 24-h urinary excretion of catecholamines and their metabolites were all increased (Table 1). The adrenal cortical hormone seemed to be within the normal range, but the 1-mg dexamethasone suppression test revealed mild autonomous cortisol secretion (5.9 μg/dl). Contrast-enhanced computed tomography (CT) revealed a cystic retroperitoneal tumor with a maximum diameter of 88 mm within the right adrenal gland (Fig. 1a). The tumor included a walled nodule, revealed by its contrast effect in the early phase. The interior of the cyst was filled with a low-density fluid with no observed contrast effect. On magnetic resonance imaging (MRI), the nodule exhibited a low signal in the T1-weighted image and a high signal in the T2-weighted image. The cyst wall accumulated an abnormal level of the marker in both 123I-MIBG scintigraphy and positron emission tomography with 2-deoxy-2-[fluorine-18] fluoro-D-glucose/CT (Fig. 1b, c). From these findings, although the 24-h urinary excretion of catecholamines and their metabolites were not sufficiently high enough to meet the diagnostic criteria of pheochromocytoma, in consideration of image findings and clinical course, we strongly suspected that the tumor was predominantly a degenerating pheochromocytoma. The patient was scheduled to undergo tumor resection.

**Table 1:** Findings of catecholamines and their metabolites, and adrenocortical hormone

	Value		Reference ranges
	Preoperative	Postoperative	
Plasma concentration			
Adrenaline	0.12 ng/ml	0.03 ng/ml	< 0.10 ng/ml
Noradrenaline	0.42 ng/ml	0.37 ng/ml	0.10–0.50 ng/ml
Dopamine	0.02 ng/ml	0.02 ng/ml	0.03 ng/ml
Cortisol	5.9 μg/dl	–	4.5–24 μg/dl
Renin	4.1 ng/ml/h	–	0.3–5.4 ng/ml/h
Aldosterone	11.2 ng/dl	–	3.0–12 ng/dl
24-h urinary excretion			
Adrenaline	93.5 μg/day	24.7 μg/day	3.4–28.9 μg/day
Noradrenaline	269 μg/day	134 μg/day	48.8–168 μg/day
Dopamine	1600 μg/day	780 μg/day	386–981 μg/day
Metanephrine	0.39 mg/day	0.06 mg/day	0.04–0.18 mg/day
Normetanephrine	0.43 mg/day	0.24 mg/day	0.08–0.33 mg/day
Vanillylmandelic acid	6 mg/day	2.8 mg/day	1.5–4.3 mg/day

However, preoperative MRI incidentally revealed a cerebral aneurysm, 8 mm in diameter, at the junction of the basilar and superior cerebellar arteries (Fig. 1d). We, therefore, discussed with a neurosurgeon, anesthesiologist, and endocrinologist whether the retroperitoneal tumor or the cerebral aneurysm should be prioritized for treatment. Based on its size and location, the annual rupture rate of this cerebral aneurysm was estimated to be approximately 1% [3], and its deep location and large size posed a relatively high surgical risk. We, therefore, decided to proceed with the resection of the tumor, aware of the risk of an intraoperative rupture of the aneurysm subsequent to surgery-induced hypertension.

We decided to conduct more rigorous blood pressure management for the patient’s surgery. Her preoperative blood pressure was managed under 120 mmHg using 32-mg/day doxazosin and 2.5-mg/day carvedilol. We used epidural anesthesia for thorough analgesic management and general anesthesia centered on propofol and remifentanil, and we carefully monitored the blood pressure. After inducing general anesthesia, a lumbar catheter was placed to monitor for intraoperative rupture of the cerebral aneurysm. In addition, we used transcranial motor-evoked potential and somatosensory-evoked potential monitoring to track her intraoperative neurological function. Subsequently, the surgical procedure was performed via a right subcostal incision with upper midline extension. During the surgery, we focused on fluctuations in blood pressure. Prior to full-scale tumor mobilization to prevent excessive secretion of catecholamine, we first ligated and separated the feeder arteries, consisting of three right adrenal arteries, followed by the drainage vein, consisting of a right adrenal vein. Next, we removed the tumor with minimal mobilization. The tumor had not noticeably invaded the surroundings. There was no blood outflow from the lumber catheter during the procedure. We withdrew the catheter immediately post and used head CT to confirm there had been no intracranial hemorrhage. No intraoperative blood pressure fluctuation was observed, and the cerebral aneurysm monitoring devices showed no abnormality. Complete resection (R0) was achieved by pathologically determining the negative surgical margin during surgery.

**Fig. 2:**
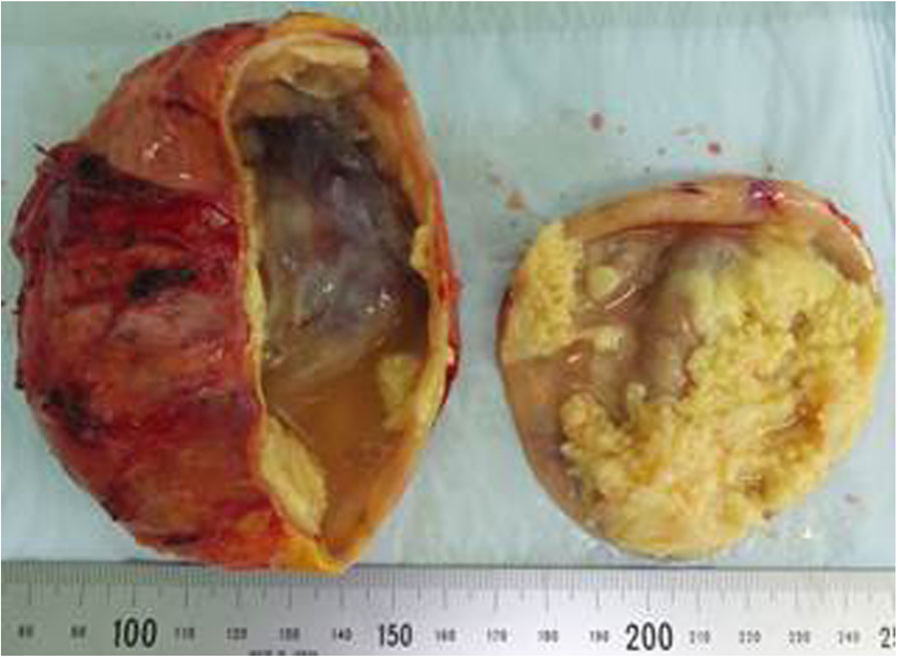
Macroscopic finding. Macroscopically, the cystic tumor was approximately 100 mm in diameter with an interior that was almost necrotic and filled with turbid brown liquid

**Fig. 3:**
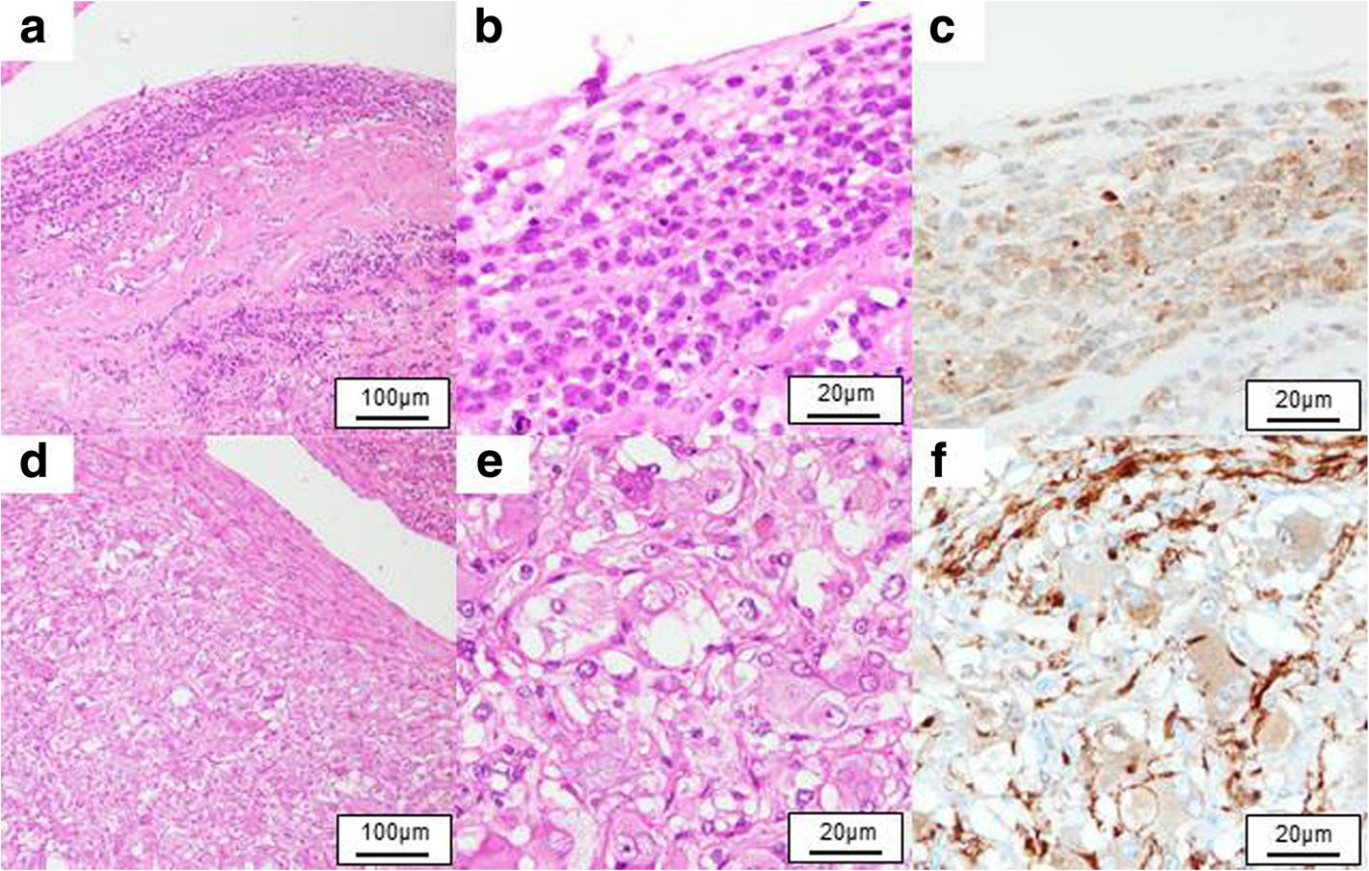
Microscopic findings. Microscopically, two types of atypical cells with enlarged heterozygous nuclei are observed in a part of the cyst wall. **a–c** Neuroblastic cells immunohistochemically positive for neuron-specific enolase [**a** hematoxylin/eosin (HE), × 100 magnification; **b** HE, × 400 magnification; and **c** neuron-specific enolase, × 400 magnification]. **d–f** Cells morphologically similar to ganglion cells and immunohistochemically positive for S-100 (**d** HE, × 100 magnification; **e** HE, × 400 magnification; and (**f**) S-100, × 400 magnification)

Macroscopically, the cystic tumor was approximately 100 mm in diameter with an interior that was almost necrotic (Fig. 2). Microscopically, two types of atypical cells with enlarged heterozygous nuclei were observed in a part of the cyst wall (Fig. 3): neuroblastic cells positive for neuron-specific enolase (Fig. 3a-c) and cells morphologically similar to ganglion cells and positive for S-100 (Fig. 3d-f). The ganglion cells were also immunohistochemically positive for tyrosine hydroxylase, DOPA decarboxylase, dopamine-beta-hydroxylase, and phenylethanolamine-N-methyltransferase. These two types of atypical cells were observed to form nodules without intermixing. Because no tumor cells judged as pheochromocytoma were observed, the tumor was histopathologically diagnosed as nodular GNB within the right adrenal gland. In addition, because the position of the tumor cells coincided with the site where 123I-MIBG scintigraphy showed abnormal accumulation, the tumor was diagnosed as a catecholamine-producing GNB.

The postoperative course was good, and the patient’s blood pressure after surgery was maintained under 110 mmHg with no antihypertensive agents. She was discharged on the 7th day postoperatively. An examination after discharge confirmed that her blood and urinary catecholamine levels and metabolite excretion had returned to normal (Table 1). Three months postoperatively, no recurrence was observed.

### Discussion

GNBs frequently occur in children, but their onset in adults is extremely rare [4]. Adult GNBs have a high potential to be malignant, and there have been many reports of distant metastasis to various organs, including the liver and bones [5]. Most reported adult GNBs do not produce catecholamines; therefore, catecholamine-producing GNBs are considered to be particularly rare [6]. In our case, GNB developed in the adrenal gland. Including this case, there have been only 14 case reports in the English-language literature on GNB in adult adrenal glands [4–16], except for composite types with pheochromocytomas. Among these 14 cases, only four (including the present case) involved catecholamine-producing GNBs [6–8] (Table 2). Hypertension has been reported in approximately 10% children with neuroblastoma [2], but only one other case of an adult GNB patient with hypertension has been reported [8]. In that case and ours, the 24-h urinary excretion of catecholamines and their metabolites showed abnormally high levels, suggesting a relationship between the tumor and hypertension. It is difficult to distinguish a catecholamine-producing GNB complicated with hypertension from a pheochromocytoma based on catecholamine dynamics, such as the 24-h urinary excretion of catecholamines and their metabolites, or 123I-MIBG scintigraphy. We, therefore, assumed that the tumor was a pheochromocytoma preoperatively. However, because both pheochromocytomas and GNBs are classified as malignant tumors, it was appropriate to resect the tumor.

**Table 2:** Cases of catecholamine-producing ganglioneuroblastomas

First author (year)	Age (years)	Sex	Urinary excretion	Tumor size (cm)	Hypertension	Treatment	Metastasis	Survival
Cameron (1967) [6]	58	F	Ad↑, NAd↑, Dopa↑, VMA↑	(−)	(+)	Surgery	(−)	3.5 years, alive
Koizumi (1992) [5]	47	F	Ad↑, NAd↑, Dopa↑, M↑, NM↑, VMA↑	9	(−)	None	Bone marrow	3 months, died
Sargazi (2006) [4]	45	F	Dopa↑	(−)	(−)	Surgery, ^131^I-MIBG	Liver, neck	5 years, alive
Current case	73	F	Ad↑, NAd↑, Dopa↑, M↑, NM↑, VMA↑	10	(+)	Surgery	(−)	3 months, alive

*F* female, *Ad* adrenaline, *NAd* noradrenaline, *Dopa* dopamine, *M* metanephrine, *NM* normetanephrine, *VMA* vanillylmandelic acid, ^*131*^*I-MIBG* ^131^I-meta-iodobenzylguanidine

It was also noteworthy that our case had a cerebral aneurysm. A catecholamine-producing tumor with a cerebral aneurysm requires much more cautious management, but there is no definite consensus over management because of limited reports. A search of PubMed found no previous reports of GNB with a cerebral aneurysm, although there have been five reported cases of pheochromocytoma with cerebral aneurysms [17–21]. In four of these cases, treatment of the tumor preceded the treatment of the cerebral aneurysm [17–20], even though in all four cases, the risk of rupture was very high or there was a history of rupture. One of these four cases developed cerebral infarction during the cerebral aneurysm surgery. In the fifth case, treatment for the pheochromocytoma proceeded because of the low risk of rupture [21]. In all of these cases, the focus during surgery was only on fluctuations in blood pressure. In the present case, we thoroughly monitored for a potential rupture using an intraoperative lumbar catheter, monitored somatosensory and motor-evoked potentials to track neurological function, and performed a head CT immediately after surgery. Even though the risk of spontaneous rupture of the cerebral aneurysm was low, the intraoperative blood pressure fluctuation owing to the catecholamine-producing tumor had the potential to affect the status of the aneurysm. If any of these intraoperative monitoring processes suggested an aneurysmal rupture, we would have performed an immediate craniotomy to repair it. If the tumor surgery was in its final stage, we would have performed a craniotomy after completing the resection; conversely, if the feeding arteries had not been treated and the tumor surgery was still at an early stage, we would have given priority to the craniotomy.

For catecholamine-producing tumors with a cerebral aneurysm, whether the tumor is pheochromocytoma or GNB, careful consideration should be given to the order in which the treatments should take place, taking into consideration the risk of rupture and the possibility of malignancy. Our strategy for this catecholamine-producing tumor with cerebral aneurysm may be helpful in the treatment of similar cases in the future.

## Conclusions

We reported here the case of an adult patient who developed an extremely rare catecholamine-producing GNB with a cerebral aneurysm. Such a case required careful consideration of the order in which the tumor and cerebral aneurysm should be treated. During tumor resection, careful perioperative management was important. Further similar cases should be accumulated to establish a consensus on the treatment strategy.

## Abbreviations

CT: Computed tomography
GNB: Ganglioneuroblastoma
MIBG: Meta-iodobenzylguanidine
MRI: Magnetic resonance imaging
